# Sanitary Legislation a First Duty of a Government

**Published:** 1857-07

**Authors:** Benjamin Daniel


### SANITARY LEGISLATION A FIRST DUTY OF A GOVERNMENT.

By BENJAMIN DANIEL, M.R.C.S., L.S.A.

THAT the improvement of our existing social condition is the knotty problem of this age; that hygiene is an agent of extensive power in its influences upon the physical and moral conditions of a people; and that it is the only expedient by which the crowded inhabitants of cities can be maintained in health and vigour,—these are strong reasons why sanitary legislation should form a first duty of a government.

It says little for real progress, that, after eighteen hundred years of Christianity, we should still be asking the old question: How much of the disease and vice we see surrounding us, depends upon our exclusive devotion to material prosperity, and on our habitual disregard of humanity, save as so much working material of fluctuating value, to be regulated by the law of supply and demand? Our large towns are filled with hospitals for the sick, with juvenile reformatories, female penitentiaries, and adult criminals' aid societies. There is an ointment for every sore. Meanwhile the weeds in our garden of civilisation grow on apace, faster than our gardeners can root them out. They threaten to overrun our most daintily cultivated flowers, and to appropriate for themselves those elements of the soil which, without the protection of the vigilant horticulturist, the fairer and tender plants cannot maintain for themselves.

There is no class whose pursuits more frequently call them into the presence of social evils than that which is engaged in the profession of medicine. There are no classes of men more fitted to analyse these results, and to trace them to their origin, than they who are habitually engaged in tracing diseases to their source.

The vocation of the physician is not that which excited the ridicule of the humorists and satirists of bygone days. He need no longer be ashamed to confess the impotence of his art to remove what is the opprobrium of society, namely, the diseased products of want and misery on the one hand, and of wealth and luxury on the other. How frequently does it occur to the observing physician, to regard the human suffering which comes before him with feelings of distress, if not of despair! How frequently does he lament the inability of his art, and the impossibility of eradicating morbid results, the seeds of which have long since been intermingled and sown broadcast among the very germs of life!

Registrars' returns inform us that not half the children born alive attain the age of twenty years. An average obtained by assurance societies, gives the following results:—

Of 1000 living children born, there remain alive—

| | | | |
|---|---|---|---|
| At 10 years | 534 | At 50 years | 307·5 |
| 20 „ | 485 | 60 „ | 229·9 |
| 30 „ | 424 | 70 „ | 133·6 |
| 40 „ | 370 | 80 „ | 44·7 |
| | | 100 „ | 1·2 |

Of 10,000 living children born in England, only 8,461 are alive at the end of the year; and of the deaths the poor of the large towns contribute the greater share. Thus, as Hamlet might say, we have a massacre of innocents every day in the year, if we could but see it. Despite of our virtues, our magnanimity, and our power, civilisation nevertheless extorts from us the sacrifice of its holocausts of silent victims.

But physical evils are not the only ones which demand the medical man's attentive consideration. There is a crop of moral evils which grow on a physical soil, and which can only be remedied by physical means. The truth is, we must not be of those who rob the widow of her mite, and for pretence make long prayers. Religious exhortations, unaccompanied by good works, will never effect much real improvement amongst the masses. The question is asked—What is to be done with our criminal population? The culprits, by their increasing numbers, are becoming unmanageable. Society, too, has some compunction about chastising its illegitimate offspring, and would persuade them to be good ; if not, society has no alternative but to shoot, hang, or imprison the malefactor. In the meantime, it is a proper question to ask—How much of the causes of depravity and vice are within the control of legislation ? Are the ruling classes, while they are professing the public good, really availing themselves of every known means of elevating the moral standard of the people whom they govern ? It is an incontrovertible fact, that the result of adverse circumstances will be impressed on the physical organisation. Crime has a physiognomy of its own, with a forehead villanously low. This faulty organisation, I have no hesitation in saying, is, in numberless cases, a simple result of external influences. And these influences, whatever they may be, are capable of modification by good or by bad government.

How comes it that we have so disregarded the sacred writings of the first dispensation ? Is there no meaning in those minute sanitary regulations which abound in them ? I am afraid that in our overweening self-complacency, we are apt to think too much of our own wisdom and philosophy, and to neglect many simple lessons of antiquity, of which bitter experience alone can teach us the value. In these ordinances the sanitary reformer perceives a recognition of the high importance which the physical constitution occupied in the divine law ; and daily experience of the intimate alliance existing between our moral and physical state, proves how far-seeing was that legislation, which, from the cradle, inculcated on the nation sanitary precautions and cleanliness, as essential parts of its religious duties.

In all laws, which have for their aim human improvement, man must be considered as a whole. It will not do to cut him up into sections, material, spiritual, or intellectual, or to develope any one part at the expense of those which remain.

It must not be objected to the medical reformer, that he too strongly insists on the moral necessity of arrangements conducive to bodily welfare, and that his views are materialist ; for while his daily experience shows him the constant action and reaction between the mind, the body, and the emotions, he is open to confess his utter ignorance of their essential nature. The chemist can manipulate his materials, can analyse or combine, but he can only theorise as to the rationale of the causes of the results. He cannot either form or destroy an infinitesimal atom. However he may endeavour to fix it, it will speedily assume one form after another. Matter in every shape is as incomprehensible as mind, and its changes and phenomena are as subtle and mysterious. It is the true Morpheus, an ever-changing indestructible essence, wandering through the physical world, at one moment forming an integrant particle of the chalky cliffs of old Albion, at another showing the "metal of her pasture," in the bones and the sinews of her sailors. We can follow the circulation of particles of matter, even in the "sweet south-west wind breathing on a bank of violets, stealing and giving odour." It is not for us to seek to dogmatise on the hidden mysteries of nature. We have nothing to do with theories. Common observation teaches us that, under favourable auspices, humanity thrives and becomes the paragon of animals, whilst, under the pressure of adversity, it descends lower and lower in the scale of degradation.

It is no arrogant assertion to say that a knowledge of the laws of nature enables us to modify organisation. Such an application of science is made extensively available by the breeders of horses, oxen, sheep, etc. If as much care were bestowed on the breeding of men as upon the breeding of cattle, we should not have books written on the degeneration of the human race.

It is a well known fact, that the condition of the labouring classes in towns, if not in the country, is pregnant with the sources of disease. Consumption, scrofula, fever, reckon their victims by thousands. Nor are the wealthier classes exempt from their peculiar ailments; but these, such as heart-disease, dropsies, apoplexies, diseased livers, etc., are rather the consequences of repletion than of exhaustion. It is stated on French authority, that the artisans of towns become extinct in the third generation. The manner of life in manufacturing and commercial towns is so injurious to their population, and the drain upon life so exhausting, that were it not for the constant immigration of fresh blood from the country, it would gradually be used up. How is it that while we are pursuing wealth and power we should be neglecting our population, or be seeking to rid ourselves of the most vigorous part of it by extensive emigration? In the late war men were scarce; we had to recruit and form bands of foreign mercenaries, whilst we allow half our children to die in infancy, and our people to be decimated in their dwellings by preventible diseases. Yet authority is not wanting to show that we are thus pursuing a suicidal policy. Lord Bacon says:—"Neither is money the sinews of war (as it is trivially said), when the sinews of men's arms in base and effeminate people is failing, . . . as for mercenary forces, which is the help in this case, all examples show, that whatsoever estate or prince does rest upon them, he may spread his feathers for awhile, but he will mew them soon after."

Sanitary reform, otherwise the physical improvement of the nation, thus broadly stated, is synonymous with real social progress, and constitutes a policy alike radical and conservative. It offers a solution of many difficulties and dangers which beset established institutions. The author already quoted says:—"As for discontentments, they are in the politic body like humours in the natural, which are apt to gather preternatural heat and inflame." It may, however, be truly stated, that humours in the natural body do really beget political discontentments. St. James asks:—"From whence come wars and fightings among you? come they not hence even of your lusts that war in your members?"

Society is the aggregate representative of individuals. It may be likened to the liver, which is an agglomeration of small livers; to a muscle, which is composed of myriads of smaller muscles—fibres and fibrillæ, each of which has its own proper and inherent power of contractility. When unanimous, they all pull together and exert their influence on one common tendon. Should anything interfere with the regular action of these minute organs, the whole machine goes wrong. Their functions are performed painfully and irregularly. When the health of the human body is impaired, it gives rise to many morbid cravings, and excitement becomes a necessity to extinguish physical and moral discomfort. Montaigne speaks of a soldier so remarkable for bravery, as to attract the notice of an emperor, who, on speaking to him, and finding him troubled with a painful and miserable disease, immediately charged his own physician with his cure; but no sooner were his health and comfort restored, than he quitted the profession of arms, his life being more precious to him now that he had some pleasure of his existence. It is a bad thing for a state when the pressure upon the people is so great, that the healthy requisites of existence are wanting to them. Who are the men of barricades? Not the well fed, warmly clad, snugly housed citizens. But they are the rabble of the *fauxbourgs;* dirty, ill fed, houseless vagabonds, like Falstaff's army, fit food for powder only. Comfortable people have something to lose, and are not over fond of fighting. Have not urgent private affairs superseded all other considerations of honour, glory, or patriotism, when the character of our country has been at stake?

Sanitary science may be unnecessary among a healthy vigorous people, inhabiting a wide and open country, or living amid the natural glories of mountains and valleys, woods and streams. Such an existence is compatible with the highest physical perfection. It is, however, quite a different thing when we have to deal with a people immured in brick and mortar, earnestly pursuing wealth, or seeking a livelihood at the expense of life itself. It is sanitary science, founded on the laws which regulate health, that calls upon the government to adopt measures which nature itself teaches to be necessary for the proper development of humanity. Where, in the existing state of society, radical reforms cannot be effected, it suggests the means by which existing injurious influences may be alleviated or counteracted. With other things, observation teaches us, that variety of employment is necessary to prevent a man from degenerating into a mere machine; hence the necessity for shortening hours of labour, and permitting an opportunity for intellectual and moral improvement. Particular exercises develope particular faculties. Excellence will be found, however, in a well maintained equilibrium between the mind, the body, and the passions. All else is extravagance and monstrosity. Athletes of various kinds are specimens of exaggerated development, and show the effect of training, but they are not to be regarded as samples of civilisation. We place in the same category all intellectual giants, who pursue the development of the faculties of the mind at the expense of the body, and very often of the mind itself. How many geniuses have there been whose genius has carried them to the verge of insanity? The laws of life teach that each individual may be endowed, as it were, with a certain amount of power; that in using it we should act economically, and not draw upon it too much in one direction, seeing that what we gain one way we are likely to lose in another. A great deal depends upon education and habits, and these are, more or less, dependent upon good or bad laws. The pleasures of science and of the imagination have been dilated upon by lordly lecturers; but what absurdity it is to expect, when a man's life alternates between the hovel and the workroom, that he should forget his actual miseries, revel in transcendent dreams of happiness, or plume himself on the dignity of humanity. The study of philosophy, and the curiosity which inquires into the essence of our nature, are pleasing to a man who can sit in a well stuffed morocco-lined easy chair, in a quiet and snug library. It may afford him intellectual gratification sweeter than the daintiest luxuries of the palate. But a man, surrounded by the *res angusta domi*, at his wits' end to get himself and family bread, is not in a fit state for such quiet and comfortable enjoyment. Before intellectual or moral improvement can be expected, the pressure of existence must be made less, and some of the more glaring physical impurities which beset the poorer classes must be removed.

In English papal times, the church probably owed some part of its efficiency to its extensive almsgiving. I believe that if the religious system of relieving the poor, which prevailed in monastic times, could be compared with that which has, from the reign of Queen Elizabeth to the present day, been pursued by our municipal authorities, the latter would gain nothing by the comparison.

The physical regeneration of the people is a necessary basis of all moral and intellectual improvement. I cannot understand how a healthy mind in a healthy body is to be expected, when the working classes, from their poverty, are crammed into over-crowded dwellings, the air of which reeks with uncleanness, while the inmates are allowed, if not obliged, to feed on food and drink compounded of the most abominable adulterations.

Is there any wonder that creatures existing, and children begotten and reared, under such circumstances, should have little respect for our social institutions? Can it be doubted that this degrading poverty is the fruitful source whence our gaols, hospitals, and workhouses are filled to overflowing? There is a class continually increasing in numbers, to whom may be said—

> "The world is not thy friend, nor the world's law."

It was the first Bonaparte who said that European wars were social wars. He despised the *ideologists*, and considered the sword the readiest solution of social difficulties.

The policy of the government of every country will always be the social amelioration of the people; and to this there is no auxiliary more important than that which, in relieving the burdens of the people, makes the healthy requisites of existence more accessible to them, while, at the same time, it teaches them to avoid that which is pernicious, and select that which conduces to their physical welfare.

